# Report on the Military and Medical Organization of the Reeducation Centers for Soldiers Evacuated from the Front

**Published:** 1918

**Authors:** 


**Report on the Military and Medical Organization of the Reeducation Centers for soldiers Evacuated from the Front.** Médecin-Major CHEVALLIER read a paper describing the French orthopedic organization and commenting on the American.

On leaving hospitals or physiotherapeutic dépôts, or on returning from sick leave, soldiers who are suffering from difficulties in function consequent upon lesions of the articulations, adherent scar tissue, muscular atrophy, rheumatism, etc., as well as those who are suffering from depression or general fatigue, must go through a course of re-education if they are to regain as quickly as possible their moral and physical health. This object is attained in the French army by different methods accordingly as the men have been wounded or ill.

Category $C^1$ consists of men who have functional difficulties which necessitate the application of special methods of physical reeducation.

Category $C^2$ comprises men who are in too delicate health generally to be able to carry out immediately the course of exercises designed for the higher category.

For Category $C^1$ there is special apparatus, such as counterpoised machines for flexion and extension, inclined planes for progressive working of the joints of the lower limbs, various devices for strengthening the wrist and fingers. These patients also perform general physical exercises requiring moderate effort. Military instruction is reduced to the minimum.

Category $C^2$ are required to march from 4 to 8 kilometres, with equipment but no knapsack. The men go through educative exercises and games arranged for walking, climbing, jumping, lifting, etc. Each lesson is interrupted by frequent breathing exercises, for many of the men have suffered from chest affections or gas poisoning.

Category $C^3$, into which the other two merge on improvement, undertakes marches of from 8 to 15 kilometres, with equipment and increasingly heavy knapsack.

The program of re-education is established every fortnight, in equal stages; at the end of each the men are examined and either maintained in their category, passed to the higher one, or removed altogether as unfit.

By these means the number of unfit men in the French army has in the space of a few months, been reduced by five-sixths.

Dr Chevallier expressed his appreciation of the work done at the Orthopedic Centre of the American army. Deformities of the feet, after having been carefully classified, are there corrected by straightening arrangements in the boots and special exercises. Each man has two pairs of boots which he changes every alternate day. The boot is always an inch longer than the wearer's foot, if correction of hammer toes is necessary. The straightening process is carried out by means of strips of leather placed transversely under the sole and behind the tuberosity when there is crowding of the metatarsus. If there is treading over on the outside of the foot the heel is raised progressively from the inside edge to the outside, and *vice versa*. Deformities of the sole under the instep are corrected by little bridges. Turned-out feet are treated by walking exercises with the toes turned in.

The results obtained are so interesting that, in Dr. Chevallier's opinion, such orthopedic centres are indispensable to every army.

